# Global perceptions of plastic surgery, suturing, and wound care among undergraduate medical students: A systematic review

**DOI:** 10.1016/j.jpra.2025.02.013

**Published:** 2025-02-26

**Authors:** Shubham Gupta, Jennifer Luu, Lakshya Sharma, Jahnavi Kalvala, Ben H Miranda

**Affiliations:** a Barts and the London School of Medicine and Dentistry, Queen Mary University of London, London, E1 2AD, United Kingdom; b Faculty of Biology, Medicine and Health, Medicine, University of Manchester, Manchester, M13 9PL, United Kingdom; c Faculty of Life Sciences & Medicine, King's College London, London SE1 1UL, United Kingdom; d School of Medicine, University of Nottingham, Nottingham, NG7 2UH, United Kingdom; e St Andrew's Centre for Plastic Surgery & Burns, Broomfield Hospital, Chelmsford CM1 7ET, United Kingdom; f St Andrew's Anglia Ruskin (StAAR) Research Group, Faculty of Health, Education, Medicine, and Social Care, Anglia Ruskin University, Chelmsford CM1 1SQ, United Kingdom

**Keywords:** Medical student perceptions, Education perceptions, Plastic surgery, Undergraduate curriculum, Suturing, Wound care

## Abstract

Plastic surgery is an important and diverse specialty in which undergraduates have an opportunity to learn important principles that are relevant to multiple specialties, including wound assessment and dressing management, suturing, burns management, craniofacial abnormality treatment, and cancer resection and reconstruction. Implementing undergraduate extracurricular learning events to supplement the already crowded undergraduate curriculum should be guided by the core knowledge requirements for medical students and the areas of weakness in their understanding. This systematic review aimed to provide a synthesis of current students’ perceptions of plastic surgery and the pedagogical methods used to teach it globally, with a focus on the UK, as the first steps in developing suitable teaching resources. The 22 cross-sectional survey-based studies retrieved from a systematic PubMed search between 28/10/12 and 13/03/24 encompass students’ opinions of plastic surgery, their attitudes of its place in medical school curricula, and their confidence in suturing and wound care as a proxy for their interest in plastic surgery. The literature is largely unified: plastic surgery is often misperceived by medical students despite general curiosity about the specialty. Methodological similarities were identified in some studies, which enabled comparisons, but only a few studies held deeper insights into the specific perceptions of students and offered implications for future practice. Small samples of chronological cross-sections and a bias towards North American study populations were noted, limiting the generalisability of findings. Recommendations to increase plastic surgery exposure in the undergraduate curriculum are suggested.

## Introduction

The breadth of knowledge gained at medical school equips junior doctors with the skills needed to perform basic effective assessment of patients, including making referrals to niche specialties such as plastic surgery in the UK National Health Service.^1^^,^^2^ Referral delays and untimely, inappropriate interventions may increase medical costs and disease burden, including complications; as such, effective training is paramount.^3^

The National Undergraduate Curriculum in Surgery offers guidance on essential plastic surgery knowledge for doctors; however, an overcrowded undergraduate curriculum of competing learning outcomes poses challenges to students acquiring such knowledge, leading to students seeking extracurricular initiatives to fulfil their educational requirements.^4^^,^^5^ The updated 2023 publication reflecting recent developments in UK medical undergraduate education contains fewer learning objectives in plastic surgery than the last curriculum in 2015, including removal of the association of plastic surgery with wound care and suturing.^3^^,^^6^

Peer-led extracurricular events may reinforce surgical learning outcomes for delegates but should be guided by the evidence base to foster optimal effectiveness.^4^ The challenge of disseminating effective knowledge of plastic surgery to medical students is further compounded by outdated stereotypes, which continue to hinder curiosity and perpetuate misconceptions worldwide.^7^, ^8^, ^9^ Evidence-based solutions to tackling misapprehensions are insufficient, in part due to publication bias undermining the reporting of single-centre workshop initiatives. Awareness of the deficits in medical students’ perceptions of plastic surgery and its teaching may provide valuable insights for policies and interventions to tackle these issues.^8^ This systematic review aimed to synthesise the current literature on perceptions of undergraduate plastic surgery education and guide future initiatives to optimise it.

## Methods

**Figure 1:**
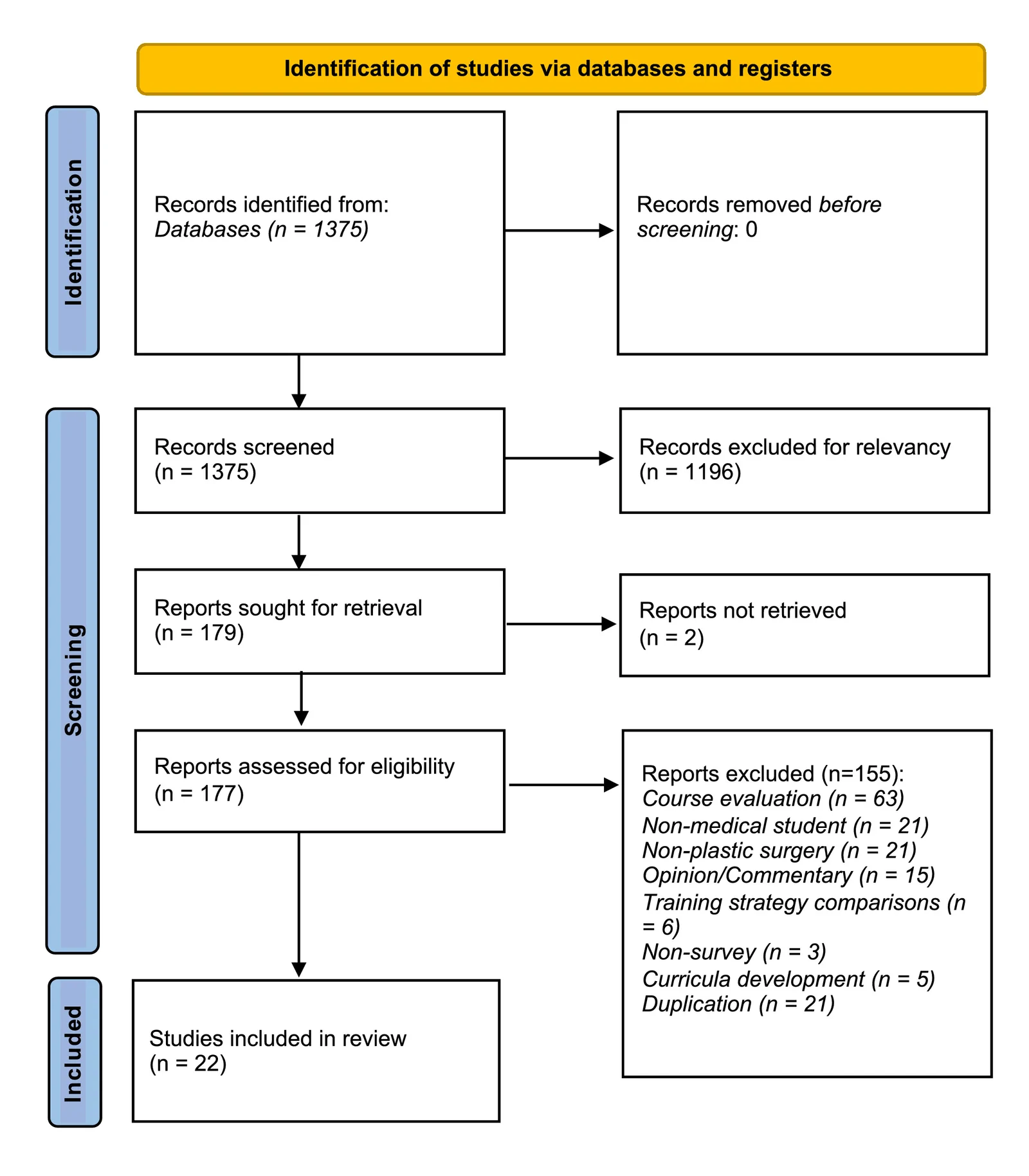
PRSIMA flowchart depicting the identification and screening process for articles including the number excluded at each stage. Non-primary research includes opinion, commentary, and systematic review articles.

A systematic literature search of PubMed was conducted for articles published within the last 10 years on 28/10/2022 and subsequently updated on 13/03/2024. Multiple search strategies (search 1: medical students + perceptions + plastic surgery + survey; search 2: medical student + curriculum + plastic surgery + survey; search 3: medical student + confidence + suturing; search 4: medical student + confidence + wound) returned a total of 1375 records (JL).^10^ Figure 1 summarises the STROBE and PRISMA guided literature search.^10^^,^^11^ After title and abstract screening for relevance based on the inclusion and exclusion criteria (Table 1), 179 records remained (SG, LS, JK). Following subsequent full-text screening for relevance, 41 records were then thematically categorised (JL, LS, JK) (Table 2) and de-duplicated, resulting in 22 included studies (Table 3) for descriptive analysis. Results relevant to the aims of this systematic review were collected on a spreadsheet (JL, SG, LS). Sample size, response rate, location, and survey domains were particularly noted to assess confidence in the conclusions (Table 3).

**Table 1:** Inclusion and exclusion criteria. Course evaluation is a publication describing a single-centre, isolated workshop or training modality using pre- and post-event survey evaluation.

Inclusion	Exclusion
• Cross-sectional study • Questionnaire-based survey • Medical students • Perception of plastic surgery as a speciality, or in the curriculum, or related skills of wound care and suturing • Literature published between 28/10/2012 to 13/03/2024 (last 10 years)	• Non-original articles and primary research • Data that did not involve perceptions collected in a survey format • Course evaluation • Non-medical students • Non-plastic surgery • Other reasons not meeting the inclusion criteria

**Table 2:** Thematic categorisation of included and excluded articles on full-text screening.

Theme	Publications categorised (n)	Publications not categorised, stratified by reason for exclusion (n)
Perceptions of plastic surgery by medical students	10	Course evaluation (7), Non-medical student (6), Non-plastic surgery (4), Non-primary research (2)
Perception of plastic surgery in the undergraduate curriculum by medical students	19	Course evaluation (15), Non-medical student (9), Non-plastic surgery (11), Non-primary research (15), Curricula development (5), No access (2)
Perception of suturing confidence by medical students	7	Course evaluation (30), Non-medical student (3), Non-plastic surgery (6), Training strategy comparisons (6), Non-survey (objective measures of suturing) (3)
Perception of wound care confidence by medical students	5	Course evaluation (11), Non-medical student (3)

**Table 3:** Study characteristics of included articles.

Reference	Locations (n)	Respondents (n); Response Rates (%)	Survey Domains
Agarwal et al.	USA (1)	230; 56.4	Medical student understanding of plastic surgery and to analyse the impact of prior plastic surgery clinical exposure on this understanding
Almeland et al.	Norway, all medical schools (4)	648; 30	State of plastic surgery in undergraduate medical training; undergraduate medical students’ level of knowledge within and attitudes towards the field of plastic surgery, answers with responses of non-medical students; predictors for a wish to pursue a career in plastic surgery
Alyahya et al.	Saudi Arabia (1)	292; N/A	Understand plastic surgery through the presentation of various clinical scenarios, identify the barriers to pursue as a future carrier, and define what are their sources of information
Austin et al.	Canada (1)	354; 35.8	Exposure to plastic surgery and student perceptions of the specialty
Aykan et al.	Turkey (1)	145; N/A	Educational internships on medical students’ perceptions of plastic surgery in correct procedure allocation
Barr et al.	USA (17)	2260; 28.5	Practical skills and confidence
Farid et al.	UK (1), and Canada (1)	243; (UK 171; 52%) (Canada 72; 54%)	Basic understanding, preferred learning method, and factors influencing a career choice in plastic surgery
Fraser et al.	Canada (1)	214; N/A	Medical students’ knowledge and perceptions of plastic surgery and factors influencing opinions
Gazibara et al.	Serbia (2)	52; 81.25	Self-perception of practical skills
Gazibara et al.	Serbia (1)	390; 77.8	Demographics, students’ self-perception on practical skills, and students’ self-perceived readiness to start working with patients
Green et al.	USA (N/A)	428; N/A	Demographics, speciality factor importance, importance of medical school experiences, influence of medical school experiences on plastic surgery interest
Higgins et al.	UK (1)	160; 57	Identification of plastic surgery subspecialties, understanding of plastic surgery, opinion of the pilot and curriculum, career preferences and gender
Isenberg et al.	USA (1)	195; N/A	Measures of proficiency and self-assessment in practical skills
Jabaiti et al.	Jordan (2)	200; sample size calculation	Knowledge of surgical procedures allocation, attitude towards plastic surgery, preference of specialization, and benefits of plastic surgery to physicians and patients
Mahalingam et al.	UK (7)	156; N/A	Students’ exposure to plastic surgery, the proportion that was timetabled within the curriculum, whether they had considered a career in this field, and which aspects of plastic surgery they considered most important
Rehman et al.	UK (5)	260; N/A	Demographics, exposure to hand surgery, confidence in assessing hand injuries
Rufai et al.	UK (16)	705; N/A	Undergraduate suture training
Sasson et al.	USA (16)	265; N/A	Demographic information, exposure to plastic surgery, mentorship, suggestions for an improved experience, and perceived impact on matching into plastic surgery
Tahiri et al.	Canada, all medical schools (13)	477; 39	Applicant details, driving force behind interest in the field, and essential character traits and competencies related to successful matching
Westermann et al.	Germany, Switzerland and Austria (36), and Great Britain (3)	2907; N/A	Measures of proficiency and self-assessment in practical skills
Yaacobi et al.	Israel, all medical schools (5)	300; 50	Knowledge and perceptions regarding the field of plastic surgery and its subspecialties, and the impact of a clinical rotation in plastic surgery on these factors
Zinchenko et al.	UK (15)	348; N/A	Burns education experience and the level of competence in assessing and acutely managing patients with burns

## Results

### Perceptions of plastic surgery

Data from Jordan, Israel, the USA, Norway, and Canada revealed a striking dissonance between student-perceived understanding and the local scope of plastic surgery.^1^^,^^2^^,^^9^^,^^12^^,^^13^ Some students appear to place a greater emphasis on the field's aesthetic component and vastly underestimate the role of plastic surgery in the treatment of peripheral neuropathies, limb surgery, and trauma.^13^, ^14^, ^15^ The influence of the media has been suggested to be at least partially attributable to this perception in the USA, Canada, and Saudi Arabia.^8^^,^^13^^,^^14^^,^^16^ Social media also has important effects, including serving as a misleading substitute for students’ limited curricular exposure to plastic surgery. This may have contributed to its perception as the least desirable speciality by a small Saudi Arabian cohort.^14^ Indeed, many medical students look to external sources to gain exposure to plastic surgery, and simple interventions such as educational events may be an effective tool in changing perceptions among students aspiring to be plastic surgeons.^5^^,^^14^

### Perceptions of plastic surgery in the undergraduate curriculum

Competing priorities place imminent stresses on the design of the undergraduate curriculum. In a survey of 16 US medical schools, only 10% (n=26/265) of students recalled plastic surgery being in the curricula.^17^ Some medical students even reported having no exposure to plastic surgery elements during their entire undergraduate education.^16^^,^^17^ Between 1986 and 2002, there was a 36% decrease in UK medical schools including plastic surgery as a compulsory element, despite a desire for further education in plastic surgery being more recently noted at 2 UK universities.^4^^,^^15^ In a Norwegian cohort, medical students were equally knowledgeable about plastic surgery referrals as non-medical students. In the study, correct allocation of presentations and procedures to the relevant speciality acted as a proxy of understanding.^12^ Limited awareness of the breadth of plastic surgery was suggested, likely resulting from the evolving Scandinavian curriculum. In a study of 156 students from 7 medical schools, over 44% of students (n=68/156) uninterested in plastic surgery were more likely to understand and consider the career if given the opportunity for further exposure.^18^ Reduced curriculum exposure may influence interest and knowledge of the specialty. However, heterogeneity in medical school curricula, including variability in parameters such as teaching methods, depth of plastic surgery teaching, and placement provision, created confounding factors and reduced the comparability of studies.^4^^,^^5^ A further complicating factor is the wide breadth of plastic surgery, such that teaching its various subspecialties often overlaps with the presentation of other specialties.^18^^,^^19^

The paucity of plastic surgery representation in undergraduate curricula has also been associated with a reduction in training applications to the specialty in Canada.^16^ Suggestions for change include enhancing guidance during problem-based learning sessions, boosting clinical rotations and shadowing opportunities, and expanding informative resources on plastic surgery and other surgical disciplines.^13^^,^^14^ Dedicated plastic surgery placements, for example, have been shown to increase student accuracy in correctly allocating procedures to plastic surgery—as a proxy for plastic surgery understanding—and to support interest in the field.^14^^,^^20^, ^21^, ^22^ A short plastic surgery clinical placement was generally preferred by medical students, but low response rates and the single-centre design of many studies limited their external validity.^4^^,^^9^ Mentorship also seems to play an important role in fostering and maintaining interest, especially when lack of facilitated exposure to plastic surgery and local plastic surgery department involvement in the medical school curriculum remain pertinent barriers.^16^^,^^17^^,^^22^

### Perceptions of suturing confidence among medical students

Suturing is a basic procedural skill that students consistently have low confidence in performing.^23^, ^24^, ^25^ With competency measured as self-evaluation of the skill, it raises a concern that only 13.5% (n=95/705) of nationally surveyed students felt capable of suturing and consequently were able to meet the UK General Medical Council's competencies for graduating doctors.^5^ However, the self-critical nature of students’ desired and actual level of competency can be misaligned; third-year students at an American university rated their skills below those achieved during testing on simulated patients.^23^ The limited likelihood of suturing in finals examinations and restricted clinical opportunities has been suggested to correspond to low confidence in suturing.^25^ Student surveys have generally reported suturing teaching quality as being below student expectations in several European and Asian countries.^5^^,^^21^^,^^24^^,^^25^

Medical students may also seek extracurricular opportunities to improve their suturing confidence. In a survey of 339 UK students, 100 (29%) reported they had paid for additional training.^5^ Students with surgical aspirations demonstrated higher levels of suturing confidence despite their limited experience.^21^ Factors that negatively impacted the self-perception of students’ suturing abilities included female sex, time constraints, and limited suturing opportunities.^23^ Repetitive practice, including early and prolonged experience, increased confidence.^26^^,^^27^ Suturing on more than 5 occasions was suggested as a threshold for improving confidence.^21^ Notwithstanding the wide variability in perceived skill level among graduates, in part due to infrequent usage and practice, these findings suggest that more can be done to improve skills in basic surgical procedures among graduating medical students.^5^

### Perceptions of wound care confidence among medical students

Wound care is a highly important skill, particularly in managing burns, which is a relatively common presentation in foundation training and specialty training.^3^ However, in one study, 91% (n=296/327) of final year students at 15 UK medical schools were not confident in their knowledge of burns management.^27^ In smaller studies of Serbian medical students, wound management and bandaging were self-evaluated to be of borderline quality at between 5 and 6 out of 10.^25^

## Discussion

The aim of this systematic review was to identify and analyse the current literature on the perceptions of plastic surgery education among medical students globally, which proved challenging. Small sample sizes and repeated cross-sectional surveys in similar locations resulted in a bias towards North American study populations, which limits generalisability. Higher-order analysis was not possible as a result. Geographical, social, and cultural contexts were confounding factors; however, the literature was largely unified: plastic surgery is often misperceived by medical students despite general curiosity about the specialty. Methodological similarities were identified in studies that enabled comparisons to develop some recommendations for improving undergraduate plastic surgery education.

### The growing deficit in plastic surgery exposure and knowledge

Although most medical students do not specialise in plastic surgery, they should be confident in making pertinent plastic surgery referrals to avoid delays in treatment and unnecessary referrals to other specialties.^1^, ^2^, ^3^ Procedure allocation- taken as an indicator of basic knowledge of the specialty - was often incorrectly performed by students.^1^^,^^7^^,^^12^^,^^14^^,^^20^ This is unsurprising, as the findings of this systematic review corroborate the conclusions of individual studies that student perceptions of the scope of plastic surgery as a specialty and career are skewed.^1^^,^^2^^,^^9^^,^^12^^,^^13^^,^^17^ Perceptions of plastic surgery are driven by the media, and more recently, social media.^1^^,^^4^^,^^7^^,^^17^ Direct exposure to plastic surgeons, in the form of mentorship or placements, reduces the impact of the media on misperceptions of the specialty.^1^^,^^4^^,^^9^ The literature also suggests that there is limited exposure to plastic surgery as part of the undergraduate core curricula and that students perceive this as inadequate curricular exposure.^4^^,^^15^, ^16^, ^17^, ^18^

### Suggestions

• The current offering of the British Association of Plastic Reconstructive and Aesthetic Surgeons (BAPRAS) Undergraduate Day and online lecture series may reach a small proportion of interested students.^28^ Dissemination of guidance to local medical student plastic surgery societies organised by dedicated students, including short guides to encourage students to be aware of the specific context of plastic surgery on social media and providing connections to local plastic surgery units, could be a more grass-roots approach to changing perceptions and increasing direct exposure.

### Indirect effects on the future workforce

Suturing and wound care, which were previously associated with plastic surgery in The National Undergraduate Curriculum in Surgery (2015), are fundamental basic procedural skills for doctors.^3^ Uncertainty in these skills could result in unnecessary referrals to the specialty and deter aspiring young plastic surgeons from applying. A lack of repeated exposure to suturing may reduce confidence and recall and therefore decrease competence.^5^^,^^23^, ^24^, ^25^ This may offer some explanation for longitudinal data from the USA that suggests that most students lose interest in plastic surgery during medical school, and the subsequent effect on decreasing applications to an already niche specialty is a concern.^16^^,^^29^ Following appreciation of research into poor undergraduate perceptions and access to plastic surgery in the USA, mentorship activities and a longitudinal suturing curriculum have been bridging awareness of the specialty.^30^^,^^31^ The number of plastic surgery applications and posts is increasing, as is the increased need for plastic surgeons, based on the recent cosmetic surgery boom.^32^^,^^33^ High competition ratios for UK plastic surgery training posts persist, with consultant posts increasing by 3% to meet BAPRAS targets, however, it is important to note that core surgical training ratios are falling.^8^^,^^34^^,^^35^ Regional UK trends in undergraduate plastic surgery teaching and the specific needs of local medical student populations remain unknown, and early exposure is important for speciality decisions.^35^

## Suggestions

• An official mentorship programme featuring plastic surgery trainees as mentors could maintain interest and exposure. It would be interesting to note if interest and exposure correlate with medical schools associated with a local plastic surgery unit.

### A call for action

Despite students’ desire to learn more about plastic surgery, including some suggestions on optimal learning methods, strategies guiding the development of extracurricular activities are lacking.^4^^,^^13^, ^14^, ^15^^,^^18^ Recent systematic reviews focusing on alternative approaches to teaching medical students and undergraduate teaching of surgical skills in UK complement the present review, highlighting growing concern about the lack of consensus to the ‘standard’ of undergraduate plastic surgery education. Trials of small-group delivery in one-day courses, optional modules, and e-learning were associated with response bias and observer-expectancy effect, and no long-term follow-up to objectively assess efficacy was noted.^35^, ^36^, ^37^ An inconsistent UK picture of plastic surgery education raises concerns on the future of meeting public health needs and the workload of colleagues.^37^ As such, the findings of this review highlight a need for research on local plastic surgery curricula design, and greater support for local extracurricular initiatives by dedicated students.

### Suggestions

• Incorporation of a short placement of 2–3 days into a trauma and orthopaedics placement as part of a larger trauma placement is suggested, albeit with a low evidence base. Anticipated issues include limited access to local plastic surgery units for some medical schools and a burdening effect of the minimal exposure to orthopaedics, as described in the literature.^38^
• To encourage awareness of plastic surgery, broad learning objectives that overlap with other surgical specialties could be reintroduced into the National Surgical Curriculum. Our suggestions include the theory of healing wounds and burns management, practical sessions on wound care, and a longitudinal exposure to suturing, such as a session at the entry point to clinical years and a refresher session prior to graduation. It is more appropriate for consultant plastic surgeons to suggest competencies, with considerations of expected details on a referral, in the style of a Delphi study, similar to the Scandinavian approach based on perceived educational deficits.^39^

### Limitations

The evidence available was limited due to heterogeneity in study design, temporality, and cross-sectional sampling, which restricted external validity. The exclusion of single-centre pre- and post-event workshop feedback (course evaluations) to restrict the effect of selection bias and response shift bias further reduced the scope of evidence. Only English-language publications in PubMed were considered, which may have provoked a selection bias towards Western medical schools.

## Conclusion

Plastic surgery is a commonly misperceived field by medical students, in part due to limited curricular exposure as well as the influence of media and social media. Differences between countries and a dearth of high-quality regional data limited the findings of this review. Due to the critical role of plastic surgery, particularly in managing trauma and burns, among other conditions, it is important that medical students are confident with the scope of plastic surgery to make appropriate referrals as doctors. Ways to improve this include incorporating short two- or three-day trauma placements into clinical surgical blocks, introducing mentorship schemes, and broadening the National Surgical Curriculum to include relevant topics. The results of this review led us to conduct a UK national survey on undergraduate perceptions and exposure to plastic surgery.^40^

## Funding

None.

## Ethical approval

None.

## CRediT authorship contribution statement

**Shubham Gupta:** Conceptualization, Methodology, Writing – original draft, Project administration. **Jennifer Luu:** Conceptualization, Methodology, Writing – original draft, Project administration. **Lakshya Sharma:** Writing – original draft. **Jahnavi Kalvala:** Writing – original draft. **Ben H Miranda:** Writing – review & editing, Supervision, Project administration.

## Conflicts of interest

None.
