# Corrigendum to “Global perceptions of plastic surgery, suturing, and wound care among undergraduate medical students: A systematic review” [JPRAS Open 44 (2025) 259-268]

**DOI:** 10.1016/j.jpra.2026.05.025

**Published:** 2026-05-15

**Authors:** Shubham Gupta, Jennifer Luu, Lakshya Sharma, Jahnavi Kalvala, Ben H. Miranda

**Affiliations:** a Barts and the London School of Medicine and Dentistry, Queen Mary University of London, London, E1 2AD, United Kingdom; b Faculty of Biology, Medicine and Health, Medicine, University of Manchester, Manchester, M13 9PL, United Kingdom; c Faculty of Life Sciences & Medicine, King's College London, London SE1 1UL, United Kingdom; d School of Medicine, University of Nottingham, Nottingham, NG7 2UH, United Kingdom; e St Andrew's Centre for Plastic Surgery & Burns, Broomfield Hospital, Chelmsford CM1 7ET, United Kingdom; f St Andrew's Anglia Ruskin (StAAR) Research Group, Faculty of Health, Education, Medicine, and Social Care, Anglia Ruskin University, Chelmsford CM1 1SQ, United Kingdom

We regret that titles were included in the authorship metadata.

We would like to apologise for any inconvenience caused.

